# The impact of smoking status on radiographic progression in patients with ankylosing spondylitis on anti-tumor necrosis factor treatment

**DOI:** 10.3389/fmed.2022.994797

**Published:** 2022-10-17

**Authors:** Bora Nam, Bon San Koo, Nayeon Choi, Ji-Hui Shin, Seunghun Lee, Kyung Bin Joo, Tae-Hwan Kim

**Affiliations:** ^1^Hanyang University Institute for Rheumatology Research, Seoul, South Korea; ^2^Department of Rheumatology, Hanyang University Hospital for Rheumatic Diseases, Seoul, South Korea; ^3^Division of Rheumatology, Department of Internal Medicine, Inje University Seoul Paik Hospital, Inje University College of Medicine, Seoul, South Korea; ^4^Biostatistical Consulting and Research Lab, Medical Research Collaborating Center, Hanyang University, Seoul, South Korea; ^5^Department of Radiology, Hanyang University Hospital for Rheumatic Diseases, Seoul, South Korea

**Keywords:** ankylosing spondylitis, radiographic progression, cigarette smoking, anti-TNF agent, spinal damage

## Abstract

**Background:**

Ankylosing spondylitis (AS) is characterized by back pain which can lead to spinal ankylosis. Anti-tumor necrosis factor (TNF) dramatically alleviates symptoms, but spinal damage can still be progressive even during anti-TNF treatment. Smoking is a one of well-known risk factors for structural damage in AS. However, it has not been confirmed that smoking can affect radiographic progression even during anti-TNF treatment.

**Objective:**

To investigate factors associated with radiographic progression during anti-TNF treatment with a focus on smoking status which is known as one of poor prognostic factors for AS.

**Materials and methods:**

We conducted a retrospective cohort study of AS patients who began the first-line anti-TNF treatment between 2001 and 2018 according to availability of smoking data. All enrolled patients were observed until the last visit, the first-line anti-TNF discontinuation, or December 2019. Radiographic damage was assessed using the modified Stoke Ankylosing Spondylitis Spinal Score (mSASSS). The mSASSS progression rate (units/year) was calculated using the baseline mSASSS, the final mSASSS during observation period, and the duration between them. Univariable and multivariable logistic regression analyses were performed to identify associated factors of mSASSS progression rate > 1 unit/year.

**Results:**

Among 459 AS patients, 185 (40.3%) patients were never smokers, 62 (13.5%) were ex-smokers and 212 (46.2%) were current smokers at baseline. Ex- and current smokers had higher mSASSS progression rates than never smokers [never smoker 0.1 (0.0–0.7), ex-smoker 0.6 (0.0–1.5), and current smoker 0.6 (0.0–1.5) units/year, *P* < 0.001]. In the multivariable logistic analysis, current smoking [adjusted odds ratio (OR) 1.69, 95% CI 1.01–2.82, *P* = 0.047] and higher baseline mSASSS [adjusted OR 1.03, 95% CI 1.01–1.04, *P* < 0.001] were associated with a mSASSS progression rate > 1 unit/year.

**Conclusion:**

Current smoking is a modifiable risk factor for radiographic progression in patients with AS on anti-TNF treatment. Quitting smoking should be strongly recommended.

## Introduction

Ankylosing spondylitis (AS) is a systemic inflammatory disease mainly affecting the axial skeleton (1). The introduction of anti-tumor necrosis factor (TNF) therapy has revolutionized the treatment of AS patients who do not respond to non-steroidal anti-inflammatory drugs (NSAIDs); anti-TNF dramatically improves the clinical symptoms of AS. Moreover, a recent study revealed that anti-TNF can slow the radiographic progression of AS (2).

The severity of structural damage is highly variable in AS; some patients develop almost no spinal structure changes over a long disease duration, whereas others have total ankylosis of the spine even in the early stages of the disease. Therefore, individualized treatment strategy is essential. And identification of patients who are likely to develop more severe structural changes is needed. Numerous attempts have been made to identify factors associated with severe radiographic damage in AS patients using diverse radiographic scales and analysis methods. Several factors including male sex (2–4), presence of baseline damage (3–12), elevated levels of inflammatory markers or high disease activity status (2–4), eye involvement (2), unaffected peripheral joints (2), and cigarette smoking (5, 6) have been reported as risk factors for radiographic damage in AS.

Among these well-known risk factors, smoking has received substantial attention since it is a modifiable lifestyle risk factor. In addition to being a risk factors of structural damage, smoking is one of the most important poor prognostic factors for AS. Previous studies have revealed that smokers have earlier onset of inflammatory back pain, increased disease activity, and decreased functional status in AS (3–11, 13–18). These negative impact of smoking on AS may be caused by diverse changes in the immune system that lead to excessive inflammation including skewing of adaptive T-cell-mediated immunity and suppressing of immune cells function (19, 20). Moreover, previous animal studies showed that smoking increased the rate of production of CD4 + T cells, which can release interleukin (IL)-17, a key player in AS pathogenesis and bone metabolism (21, 22). However, no previous study has investigated whether smoking is related to radiographic progression even during anti-TNF treatment.

Therefore, in this study, we investigate the factors associated with radiographic progression in patients with AS receiving anti-TNF treatment by focusing on smoking.

## Materials and methods

### Patient enrollment

We conducted a retrospective cohort study of AS patients who began the first line anti-TNF (Adalimumab, Etanercept, Infliximab, and Golimumab) between January 2001 and December 2018 in a single tertiary referral hospital according to smoking data availability. All patients satisfied the 1984 modified New York criteria for the classification of AS (23). Only AS patients with more than 2 full sets of spine radiographs during follow-up duration were enrolled. The index date was defined as the date of initiation of the first-line anti-TNF agent. Patients were observed until the last visit, discontinuation of the first-line anti-TNF agent, or December 2019.

### Data collection

We collected information about demographics including age and sex, disease duration since first AS-specific symptoms, history of psoriasis, peripheral joint involvement, human leukocyte antigen (HLA)-B27 positivity, use of NSAIDS, and type of anti-TNF therapy by medical chart review. The serum concentrations of C-reactive protein (CRP) and the Bath Ankylosing Spondylitis Disease Activity Index (BASDAI) score at index date were also obtained for each patient.

Smoking data were also obtained from medical records. In our daily clinical practice, we assess smoking status predominantly when patients visit the hospital for the first time, when the anti-TNF agent is initiated, and when AS disease activity is assessed for insurance reimbursement *via* interview or self-report. Patients were asked specifically whether they were current smokers, ex-smokers, or never smokers. If they were ex-smokers, they were also asked when they quit smoking.

### Radiographic damage assessment

Spine radiographs were scored using the modified Stoke Ankylosing Spondylitis Spinal Score (mSASSS). With the mSASSS, each anterior corner of the cervical spine (lower border of C2 to upper border of T1) and the lumbar spine (lower border of T12 to upper border of S1) are evaluated in a lateral view receiving a score between 0 and 3 (0 = no abnormality, 1 = erosion, sclerosis or squaring, 2 = syndesmophyte, 3 = bridging syndesmophyte). The total score ranges from 0 to 72 (24). All radiographs were evaluated by 2 musculoskeletal radiologists blinded to the clinical data. Inter-observer reliability was assessed by calculating the ICC, which showed excellent agreement (ICC = 0.95) (2).

The mSASSS progression rate (units/year) was calculated using the baseline mSASSS, the final mSASSS of the observation period, and the duration between them. In a previous study, a change of 2 mSASSS units in 2 years was defined as moderate progression (9). Hence, we considered significant radiographic progression as an mSASSS progression rate of more than 1 unit/year.

### Statistical analysis

Patients were divided into three groups according to smoking status at baseline; never smoker, ex-smoker, and current smoker group. Demographic and clinical characteristics of each group are described in a descriptive analysis and all data are shown as median (interquartile range [IQR]) or percentage values. Three groups were compared using the Kruskal-Wallis test for non-normally distributed numerical variables and Chi-square test or Fisher’s exact test for categorical variables.

The odds ratio (OR) with confidence interval (CI) was calculated to identify factors associated mSASSS progression of > 1 unit/year using univariable and multivariable logistic analyses. Variables with *P* ≤ 0.1 in the univariable analyses were advanced to the multivariable logistic regression. To avoid multi-collinearity in multivariable analysis, disease duration was chosen rather than age.

Statistical analyses were performed using SAS version 9.4 (SAS Institute Inc., Cary, North Carolina, USA) and R version 4.0.3 (R Foundation for Statistical Computing, Vienna, Austria). *P* ≤ 0.05 was considered statistically significant.

### Ethical considerations

This study was performed according to the guidelines of the Helsinki Declaration and approved by the Institutional review board (IRB) of Hanyang University Hospital (IRB file No. HYUH 2021-10-013). The need for informed consent was waived owing to the retrospective nature of the study.

## Results

### Demographic and clinical characteristics of patients

**FIGURE 1:**
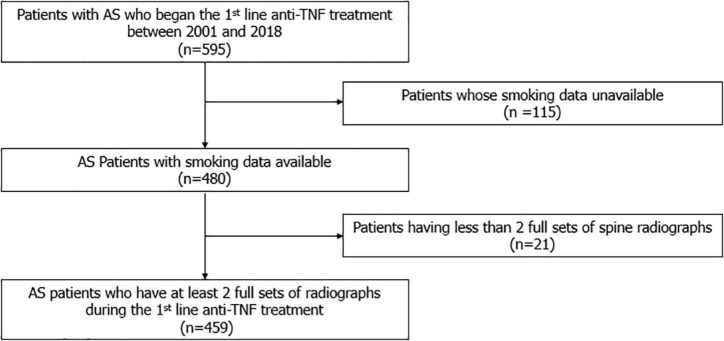
Flow chart of patient selection.

A total 459 patients with AS were included (Figure 1). The demographic and clinical characteristics of the patients at enrollment are shown in Table 1. The median age of the patients was 32.3 (27.1–39.2) years, and 88.5% were male. The median disease duration was 10.4 (4.9–15.6) years, and patients were observed for 8.0 (5.6–12.0) years. The median time interval between the baseline mSASSS and the initiation of anti-TNF agent was 5.9 (1.0–15.9) months. The median baseline and the last mSASSS during the observation period were 9.0 (5.5–23.7) and 12.4 (6.0–35.0), respectively. The median mSASSS progression rate was 0.3 (0.0–1.3) units/year, and 48.8% of the patients showed a progression rate of less than 1 unit/year. With regard to disease activity, the median CRP concentration was 2.0 (0.9–4.3) mg/dL. The median BASDAI at enrollment was 7.0 (6.0–8.0). Among 459 enrolled patients, 185 (40.3%) patients were never smokers, 62 (13.5%) were ex-smokers and 212 (46.2%) were current smokers when they started anti-TNF treatment.

**TABLE 1:** Characteristics of patients with ankylosing spondylitis.

	AS patients (*n* = 459)
Age at enrollment, median (IQR), years	32.3 (27.1–39.2)
Male sex, *n* (%)	406 (88.5)
Disease duration, median (IQR), years	10.4 (4.9–15.6)
Follow-up duration, median (IQR), years	8.0 (5.6–12.0)
Baseline mSASSS, median (IQR), unit	9.0 (5.5–23.7)
Last mSASSS, median (IQR), unit	12.4 (6.0–35.0)
mSASSS progression rate, median (IQR), units/year	0.3 (0.0–1.3)
≤1, *n* (%)	224 (48.8)
>1 and ≤ 2, *n* (%)	137 (29.8)
>2 and ≤ 3, *n* (%)	71 (15.5)
>3, *n* (%)	27 (5.9)
HLA-B27 positivity, *n* (%)	446 (97.2)
Peripheral joint involvement, *n* (%)	236 (51.4)
NSAIDs, *n* (%)	455 (99.1)
**Biologics, *n* (%)**	
Etanercept	185 (40.3)
Adalimumab	129 (28.1)
Infliximab	96 (20.9)
Golimumab	49 (10.7)
CRP, median (IQR), mg/dL (*n* = 414)	2.0 (0.9–4.3)
BASDAI, median (IQR), unit	7.0 (6.0–8.0)
**Smoking status, *n* (%)**	
Never smoker	185 (40.3)
Ex-smoker	62 (13.5)
Current smoker	212 (46.2)

IQR, interquartile range; mSASSS, modified Stoke Ankylosing Spondylitis Spinal Score; HLA, human leukocyte antigen; NSAIDs, non-steroidal anti-inflammatory drugs; CRP, C-reactive protein; BASDAI, Bath Ankylosing Spondylitis Disease Activity Index.

### Differences in characteristics of patients according to smoking status

Differences in demographic and clinical characteristics of patients according to smoking status at baseline are summarized in Table 2. Compared with other groups, the never smoker group was younger [never smoker 29.3 (23.6–38.0) vs. ex-smoker 39.4 (32.3–46.5) vs. current smoker 32.5 (29.3–37.6) years, *P* < 0.001], were less frequently male (never smoker 73.5% vs. ex-smoker 100.0% vs. current smoker 98.1%, *P* < 0.001), had lower baseline mSASSS [never smoker 7.2 (5.5–11.0) vs. ex-smoker 14.7 (6.4–31.5) vs. current smoker 10.8 (6.0–29.0), *P* < 0.001], and had more patients with peripheral joint involvement than other groups (never smoker 60.5% vs. ex-smoker 48.4% vs. current smoker 44.3%, *P* = 0.005). The median disease duration, follow-up duration, HLA-B27 positivity, medications, and baseline BASDAI and CRP were comparable between the three groups.

**TABLE 2:** Differences of characteristics of patients according to baseline smoking status.

	Never smoker (*n* = 185)	Ex-smoker (*n* = 62)	Current smoker (*n* = 212)	*P*
Age at enrollment, median (IQR), years	29.3 (23.6–38.0)	39.4 (32.3–46.5)	32.5 (29.3–37.6)	<0.001
Male sex, *n* (%)	136 (73.5)	62 (100.0)	208 (98.1)	<0.001
Disease duration, median (IQR), years	8.6 (4.0–15.4)	12.9 (5.4–19.4)	10.9 (6.0–15.2)	0.064
Follow-up duration, median (IQR), years	8.0 (5.6–12.0)	7.4 (4.4–10.9)	7.9 (5.8–12.2)	0.559
Baseline mSASSS, median (IQR), unit	7.2 (5.5–11.0)	14.7 (6.4–31.5)	10.8 (6.0–29.0)	<0.001
HLA-B27 positivity, *n* (%)	183 (98.9)	61 (98.4)	202 (95.3)	0.077
Peripheral joint involvement, *n* (%)	112 (60.5)	30 (48.4)	94 (44.3)	0.005
NSAIDs, *n* (%)	184 (99.5)	62 (100.0)	209 (98.6)	0.471
Biologics, *n* (%)				0.237
Etanercept	76 (41.1)	30 (48.4)	79 (37.3)	
Adalimumab	52 (28.1)	16 (25.8)	61 (28.8)	
Infliximab	34 (18.4)	8 (12.9)	54 (25.5)	
Golimumab	23 (12.4)	8 (12.9)	18 (8.5)	
CRP, median (IQR), mg/dL (*n* = 414)	2.2 (0.8–4.7)	2.3 (0.9–4.1)	3.1 (0.9–3.9)	0.453
BASDAI, median (IQR)	6.9 (6.0–8.0)	7.0 (5.9–8.3)	7.1 (5.9–8.0)	0.856

Non-normally distributed numerical variables are presented by median (Q1-Q3) and were tested by Kruskal-Wallis test.

Categorical variables are presented by *n* (%) and were tested by Chi-square test or Fisher’s exact test.

mSASSS, modified Stoke Ankylosing Spondylitis Spinal Score; HLA, human leukocyte antigen; NSAIDs, non-steroidal anti-inflammatory drugs; CRP, C-reactive protein; BASDAI, Bath Ankylosing Spondylitis Disease Activity Index.

### Differences in modified stoke ankylosing spondylitis spinal score progression according to smoking status

The median baseline mSASSS of the never smoker group [7.2 (5.5–10.9)] was lower than those of the ex-smoker group [14.7 (6.5–31.1)] and current smoker group [10.8 (6.0–29.0), *P* < 0.001]. There was no difference in the duration between the baseline mSASSS and last mSASSS (*P* = 0.867). However, the median of the last mSASSS of the never smoker group [8.0 (6.0–18.0)] was significantly lower than those of the ex-smoker [22.5 (10.2–42.2)] or current smoker group [18.1 (6.3–39.9), *P* < 0.001]. The median mSASSS progression rates of the never smoker, ex-smoker, and current smoker groups were 0.12 (0.00–0.71), 0.58 (0.00–1.48), and 0.55 (0.00–1.45), respectively (Table 3).

**TABLE 3:** Differences in mSASSS and mSASSS progression rate according to smoking status.

	Never smoker (*n* = 185)	Ex-smoker (*n* = 62)	Current smoker (*n* = 212)	χ^2^ (*P*)	Significant group pair*
Baseline mSASSS, median (IQR), unit	7.2 (5.5–10.9)	14.7 (6.5–31.1)	10.8 (6.0–29.0)	23.19 (<0.001)	(N vs. E), (N vs. C)
Last mSASSS, median (IQR), unit	8.0 (6.0–18.0)	22.5 (10.2–42.2)	18.1 (6.3–39.9)	29.71 (<0.001)	(N vs. E), (N vs. C)
Duration between the first and the last mSASSS, median (IQR), years	6.7 (3.9–9.4)	6.2 (3.8–8.4)	6.0 (4.1–8.7)	0.29 (0.867)	
mSASSS progression rate, median (IQR), units/year	0.1 (0.0–0.7)	0.6 (0.00–1.5)	0.6 (0.00–1.5)	18.88 (<0.001)	(N vs. E), (N vs. C)

Non-normally distributed variables are presented by median (Q1-Q3) and were tested by Kruskal-Wallis test.

*Post-hoc test using Dwass-Steel-Critchlow-Fligner method.

mSASSS, modified Stoke Ankylosing Spondylitis Spinal Score; N, never smoker group; E, ex-smoker group; C, current smoker group.

**FIGURE 2:**
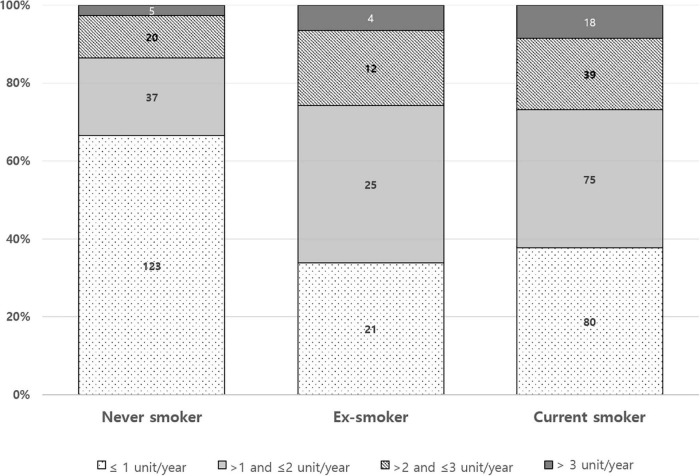
Difference in mSASSS progression rate according to baseline smoking status.

The pattern of mSASSS progression rate during anti-TNF was different according to baseline smoking status (Figure 2). The proportion of mSASSS progression rate ≤ 1 unit/year appears to be higher in the never smoker group than in the ex-smoker or current smoker group. A ratio of an mSASSS progression rate of > 3 units/year seemed to decrease from current smokers to ex-smokers to, and never smokers.

### Factors associated with significant modified stoke ankylosing spondylitis spinal score progression (modified stoke ankylosing spondylitis spinal score progression rate >1 unit/year)

To identify factors associated with mSASSS progression rate over 1 unit/year, we conducted univariable and multivariable logistic regression analyses (Table 4). In the crude analysis, disease duration [unadjusted odds ratio (OR) 1.04, 95% CI 1.01–1.06, *P* = 0.006], previous smoking (unadjusted OR 2.70, 95% CI 1.45–5.04, *P* = 0.002), current smoking (unadjusted OR 2.19, 95% CI 1.39–3.46, *P* = 0.001), and higher baseline mSASSS (unadjusted OR 1.03, 95% CI 1.02–1.05, *P* < 0.001) were associated factors of mSASSS progression rate of > 1 unit/year. And peripheral joint involvement was related to a decreased possibility of having an mSASSS progression rate of > 1 unit/year (unadjusted OR 0.59, 95% CI 0.40–0.89, *P* = 0.012). In the multivariable analysis, current smoking and higher baseline mSASSS were associated with an mSASSS progression rate of > 1 unit/year (adjusted OR 1.69, 95% CI 1.01–2.82, *P* = 0.047; adjusted OR 1.03, 95% CI 1.01–1.04, *P* < 0.001). Ex-smokers had a trend toward a higher mSASSS progression, but the values did not reach statistical significance (adjusted OR 1.93, 95% CI 0.98–3.83, *P* = 0.059).

**TABLE 4:** Factors associated with mSASSS progression rate > 1 unit/year.

Variables	Univariable analysis		Multivariable analysis	
	Unadjusted OR (95% CI)	*P*	Adjusted OR (95% CI)	*P*
Disease duration, years	1.04 (1.01–1.06)	0.006	1.02 (1.00–1.05)	0.095
Male sex	1.96 (0.95–4.02)	0.067	1.12 (0.50–2.49)	0.784
**Smoking status**				
Never smoker	Ref.		Ref.	
Ex-smoker	2.70 (1.45–5.04)	0.002	1.93 (0.98–3.83)	0.059
Current smoker	2.19 (1.39–3.46)	0.001	1.69 (1.01–2.82)	0.047
Peripheral joint involvement	0.59 (0.40–0.89)	0.012	0.76 (0.49–1.18)	0.219
NSAIDs	0.85 (0.08–9.48)	0.897		
Baseline BASDAI	0.96 (0.84–1.10)	0.530		
Baseline CRP, mg/dL (*n* = 414)	1.03 (0.97–1.10)	0.316		
Baseline mSASSS	1.03 (1.02–1.05)	< 0.001	1.03 (1.01–1.04)	< 0.001

OR, odds ratio; Ref., reference; NSAIDs, non-steroidal anti-inflammatory drugs; BASDAI, Bath Ankylosing Spondylitis Disease Activity Index; CRP, C-reactive protein; mSASSS, modified Stoke Ankylosing Spondylitis Spinal Score.

## Discussion

We investigated factors associated with radiographic progression during anti-TNF treatment by focusing smoking. There were differences in baseline characteristics of groups according to smoking status; the never smoker group was younger and had more patients with peripheral joint involvement than the ex-smoker group or the current smoker group. And the majority of female patients were never smokers. The mSASSS progression rate of our study [0.3 (0.0–1.3) units/year] seems lower than those previous noted; 1.3 ± 2.5 unit/year (9) or 0.98 units/year (25). This might be explained by the radiographic progression-relieving effect of anti-TNF on AS since only patients receiving anti-TNF treatment were enrolled in this study. Interestingly, there were differences in the baseline mSASSS and the mSASSS progression rate between groups. The median baseline mSASSS and the median mSASSS progression rate of the never smoker group were significantly lower than those of the ex-smoker group and current smoker group. After adjusting clinical factors, only higher baseline mSASSS and current smoking were found to be related to a significant mSASSS progression (mSASSS progression rate > 1 unit/year).

Smoking is one of well-known poor prognostic factor of AS. Therefore, smoking cessation is strongly recommended for AS patients. Nevertheless, we observed that a relatively large proportion of patients was current smokers (46.2%). Previous studies have shown comparable results. The prevalence of current smoking among AS patients with anti-TNF was 43% in DANBIO and 29% in the BSRBR-AS (26, 27). Considering that AS patients start anti-TNF due to high disease activity despite conventional treatment, the high prevalence of current smoking alerts us that more patients with AS continue to smoke than expected. Because of this, attention should be paid to smoking status of AS patients.

Factors associated significant radiographic progression identified in our study are consistent with those reported in previous studies. In addition to current smoking (5, 11, 13, 17, 28), baseline damage has repeatedly been associated with significant radiographic progression in AS (3–11). However, the difference between the present and previous studies is that we focused on the specific period of anti-TNF treatment continuation in AS patients. To the best of our knowledge, this is the first study demonstrating that smoking leads to radiographic progression of AS even with anti-TNF treatment using mSASSS, the most validated method for assessing radiographic damage in AS (29). Considering that smoking is a modifiable factor, our results can provide new evidence that we should encourage patients with AS to stop smoking even their disease activity seems to be well-controlled by anti-TNF treatment.

In the real-word, anti-TNF agent is used in patients with high disease activity. Patients are evaluated every 6 months and the anti-TNF agent is continued only if the disease activity is regulated. However, if the disease activity is not regulated, we begin to use of a second line anti-TNF agent or an IL-17 inhibitor (30). Therefore, we focused on a specific period of anti-TNF continuation in which the disease activity can be assumed to be controlled. However, we cannot draw conclusions about the relationship between smoking and anti-TNF response in AS patients. Further studies using cumulative disease activity or inflammation markers are needed to determine whether the increased radiographic progression arose from the genuine effect of smoking itself or from secondary changes due to excessive inflammation caused by smoking. Previous studies have shown conflict results. Smoking was associated with impaired response to anti-TNF in AS patients (26, 31), or not (32–34). One previous study reported that current and ex-smokers had shorter treatment adherence and poorer anti-TNF response than never smokers (26). On the other hand, one recent study showed conflicting results that; baseline smoking status did not affect anti-TNF discontinuation (27).

The major limitations of our study are associated with nature of retrospective studies. First, there might be a possibility of recall bias leading to misclassification of some patients since the smoking status was based on the medical records of the patients’ responses. Second, we calculated radiographic progression rate using baseline mSASSS and the last mSASSS and assumed that the rate of change is constant between them. Caution is needed in interpreting our results since that radiographic progression rate varies across individuals and also can vary within same individuals across time (35, 36). Third, Dose-dependent effect of smoking on radiographic progression was not considered. Some studies showed that the negative impact of smoking on radiographic damage was dose dependent (5, 17). Fourth, since we divided groups according to smoking status, there were differences in the baseline characteristics between the groups including sex and age. Though we performed multivariable logistic regression analyses to adjust covariates, there could be residual confounding. And relatively small sample size of ex-smoker group may reduce statistical power. In addition, only patients who had ever taken NSAIDs during follow up duration were counted for NSAIDs users. Other drugs such as disease-modifying antirheumatic drugs and steroids were not considered.

In summary, a relatively large proportion of AS patients continue smoking despite its well -known negative effects on AS. Current smoking and higher baseline mSASSS are associated with significant radiographic progression in AS patients treated with anti-TNF. Therefore, quitting smoking should be strongly recommended for AS patients under anti-TNF treatment. Our results might help improve patient care and lead to better radiographic outcomes in patients with AS since smoking is a modifiable factor.

## Data availability statement

The raw data supporting the conclusions of this article will be made available by the authors, without undue reservation.

## Author contributions

BN, BSK, and T-HK: conceptualization. BN, J-HS, SL, KBJ, and T-HK: data collecting and interpretation. BN and NC: formal analysis. BN: writing—original draft. BSK and T-HK: writing—review and editing. All authors contributed to the article and approved the submitted version.
